# Prognostic Role of Platelet-to-Lymphocyte Ratio in Hepatocellular Carcinoma with Different BCLC Stages: A Systematic Review and Meta-Analysis

**DOI:** 10.1155/2018/5670949

**Published:** 2018-08-08

**Authors:** Wan-fu Lin, Mao-feng Zhong, Yu-ren Zhang, Huan Wang, He-tong Zhao, Bin-bin Cheng, Chang-quan Ling

**Affiliations:** ^1^Department of Traditional Chinese Medicine, Changhai Hospital, Second Military Medical University, Shanghai 200433, China; ^2^Graduate School of Shanghai University of Traditional Chinese Medicine, Shanghai 201203, China

## Abstract

The role of platelet-to-lymphocyte ratio (PLR) in the prognosis of hepatocellular carcinoma (HCC) patients with different Barcelona Clinic Liver Cancer (BCLC) stages remains controversial. This systematic review and meta-analysis aimed to determine the efficacy of PLR on HCC prognosis. Five electronic databases were searched for clinical trials focusing on the role of PLR in the prognosis of HCC. A total of 297 potential studies were initially identified, and 9 studies comprising 2449 patients were finally enrolled to evaluate the association between the pretreatment PLR and clinical outcomes of overall survival (OS), disease-free survival (DFS), and event occurrence in patients with HCC in different BCLC stages. An elevated pretreatment PLR indicated unfavorable worse OS (HR = 1.73; 95% CI: (1.46, 2.04); *P* < 0.00001) and DFS (HR = 1.30; 95% CI: (1.06, 1.60); *P* = 0.01). Subgroup analysis indicated that high PLR indicated poor OS among BCLC-B/C patients without heterogeneity, while PLR in BCLC-A patients indicated high statistical heterogeneity with *I*^2^ value of 78%. As for the correlation between PLR and event occurrence, high PLR was related to poor clinical event occurrence only among BCLC-C patients, though obvious heterogeneity was observed in all different BCLC stages. In conclusion, PLR may be a significant biomarker in the prognosis of HCC in different BCLC stages.

## 1. Introduction

Inflammation, a protective immune response to harmful stimuli such as pathogens and dead cells, is mounted by the evolutionarily conserved innate immune system with tight regulation of host [1]. Homogeneous inflammation is vital for health; insufficient inflammation may lead to persistent infection of pathogens, while excessive inflammation may cause chronic or systemic inflammatory diseases. Inflammation is linked to a variety of diseases such as Alzheimer's disease, Parkinson's disease, diabetes, and cancer [2, 3]. In 1863, Virchow initially clarified the relation of inflammation and cancer through the theory of leukocyte infiltrates within tumors, which is commonly considered a hallmark of cancer now [4, 5]. Since then, more and more evidences have revealed that inflammatory response correlates closely with tumor progression such as angiogenesis and tumor invasion. It is verified that the invasion and migration of tumor cells correlate closely with inflammation-related cells, including lymphocytes [6], neutrophils [7, 8], and platelets [9]. Classically, platelets are considered as crucial effector cells in hemostasis; however, extensive experiments have also illuminated their potential role in inflammatory responses—they may recognize and kill invading pathogens and also release various mediators modifying immune and endothelial cell responses [10]. Besides, clinical studies have also implied that platelets may lead to tumor growth and metastasis [11], since higher platelet counts were associated with shorter survival time and increased recurrence after treatment in various solid tumors [12, 13]. As a crucial component of host immune surveillance system, lymphocyte plays an important role in patients with various types of malignant neoplasms [14]. Accumulated evidences indicated that tumor-infiltrating lymphocytes may influence disease outcomes of patients with malignant neoplasms [15, 16]—higher platelet counts may indicate a poor prognosis while lymphocyte infiltration around a tumor may associate with a better prognosis; the platelet-to-lymphocyte ratio (PLR) may be a useful index for the prognosis of tumor patients [17, 18].

Hepatocellular carcinoma (HCC) is the second most common cause of cancer-related deaths worldwide and is an aggressive tumor type with poor prognosis [19]. More and more evidences indicate that the occurrence and development of HCC is correlated closely with both inflammation and immunocytes [20, 21]. Recent studies also indicated that PLR may be a potential index for the prognosis of HCC after resection [22], liver transplantation [23], or transarterial chemoembolization (TACE) [24]. However, no agreement is reached among available studies due to small sample size. Thus, we conducted this meta-analysis to evaluate the prognostic role of PLR in different Barcelona Clinic Liver Cancer (BCLC) stages of HCC.

## 2. Methods

### 2.1. Information Sources and Search Strategies

Five electronic databases including PubMed, Web of Science, EMBASE, Elsevier, and Cochrane Library were searched for clinical trials in original articles. The last completed database search was undertaken on July 28, 2017. Search terms included “liver cancer,” “hepatocellular carcinoma,” “hepatoma^∗^,” “platelet,” “lymphocyte,” “platelet to lymphocyte ratio,” and “platelet-lymphocyte ratio” title/abstract. References cited in the retrieved articles were also scanned for relevant studies.

The most important inclusive criterion was studies concerning the prognostic role of PLR in HCC. Other eligible criteria include studies with data on BCLC, disease-free survival (DFS), and overall survival (OS). Nonclinical studies, case reports, review articles, editorials, comments, and articles without accessible full text for quality assessment or data extraction were all excluded.

### 2.2. Data Extraction

The selection of studies was performed independently by 2 reviewers (Wan-fu Lin and Mao-feng Zhong), and the third investigator (Yu-ren Zhang) was consulted to resolve any disagreements. The following data of each included study were collected: the information of authors such as the name and affiliation, year of publication, research time and location, sample size, patients' age, gender, BCLC stage, intervention regimes, inflammation index, time of follow-up, and outcomes of interest. We also contacted the corresponding author if we need more detailed data.

### 2.3. Quality Appraisal and Risk of Bias

The quality appraisal and risk of bias of each included study were independently evaluated by two reviewers (Huan Wang and He-tong Zhao) with Newcastle-Ottawa Quality Assessment Scale (NOS). The scale included 3 parameters of quality: selection, comparability, and outcome assessment, with ranges from 0 to 4, 0 to 1, and 0 to 3 points, respectively. The study with the highest quality may score 9 points. The study with scores ≥ 6 was considered as high quality and scores ≥ 5 as eligible quality. The consensus about the methodological quality of all the studies was achieved since any disagreement between the two reviewers was resolved through discussion.

### 2.4. Data Synthesis and Analysis

The Review Manager (RevMan, the Cochrane Collaboration, Oxford, UK) version 5.3 was used for data synthesis and analysis. To determine heterogeneity, the chi-squared and *I*-squared tests were performed. Then, fixed-effect model or random effects model was applied based on the heterogeneity of different trials. Funnel plots and Egger's tests were used to assess the potential publication bias. The main prognosis outcomes were OS, DFS, or recurrence-free survival (RFS). Pooled estimates were expressed as hazard ratios (HR) and 95% confidence interval (CI). *P* < 0.05 with two-side test was considered statistically significant.

## 3. Results

### 3.1. Search Results

**Figure 1:**
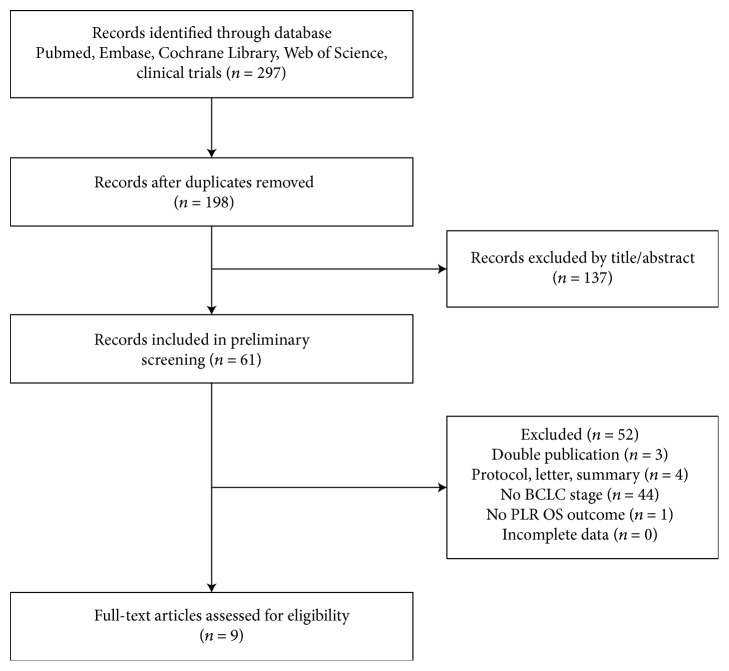
Flow diagram for study identification and inclusion.

Two hundred and ninety-seven studies potentially relevant to this research project were initially identified after searching PubMed, Web of Science, EMBASE, Elsevier, and Cochrane Library. After excluding duplicate articles, 198 potentially eligible studies were selected. Of these, 137 studies were excluded after reading the titles and abstracts. The full texts of the remaining 61 studies were carefully screened. Subsequently, 52 papers were further excluded since they did not meet the inclusion criteria. Finally, 9 studies were included in this meta-analysis. The selection process is depicted in Figure 1.

### 3.2. Characteristics of Included Studies

The 9 included studies were all published between 2015 and 2017 [24–32], among which 7 trials were from China, 1 from Japan, and 1 from Italy. All these trials were retrospective cohort studies with a total of 2449 participants enrolled. Of these included patients, 1692 received hepatic resection, 685 received TACE treatment, and 72 received sorafenib therapy. As for gender, 2122 participants were male (86.65%) compared to 327 female (13.35%), with mean age ranged from 51.98 to 67.5 years. The cases in BCLC stages A, B, and C were 966, 526, and 318, respectively, while 634 participants from 2 trials were not indicated BCLC-B/C clearly, and the remaining 5 participants were not shown BCLC stages. The median follow-up time ranged from 11.4 months to 24 months, excluding 6 studies without exact follow-up time. As for PLR, 8 studies reported a “high” PLR level with survival data, among which the cutoff value of PLR was 150 in 5 included papers and determined using different methods among the other 3 studies. The characteristics of included studies are shown in Table 1.

### 3.3. Quality Assessment and Risk of Bias of the Included Trials

The quality appraisal and risk of bias of each included review were independently evaluated with Newcastle-Ottawa Quality Assessment Scale, and the result is shown in Table 2. In the table, 1–8 represent the quality indicators from the Newcastle-Ottawa Scale: 1: is the case definition adequate? 2: representativeness of the cases; 3: selection of controls; 4: definition of controls; 5: comparability of cases and controls on the basis of the design or analysis; 6: ascertainment of exposure; 7: same method of ascertainment for cases and controls; 8: nonresponse rate.

All of the 9 papers are eligibility quality studies and 8 of which are considered to show high-quality studies (scores ≥ 6).

### 3.4. Correlation between PLR and OS

**Figure 2:**
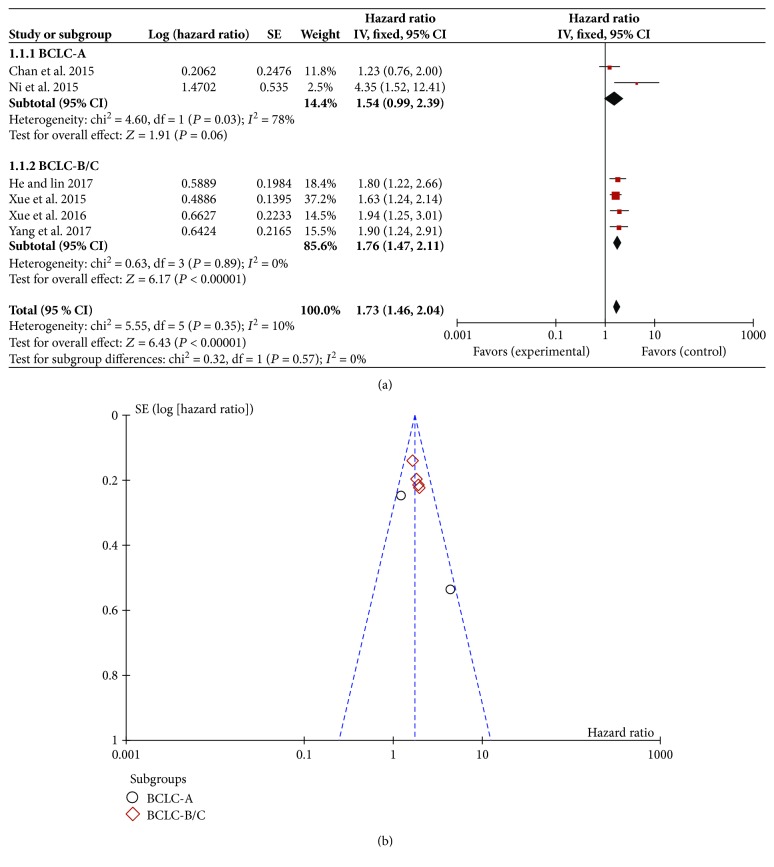
Correlation between platelet-to-lymphocyte ratio and overall survival. (a) Forest plot of comparison of the included trials; (b) funnel plot of comparison of the included trials.

Among all the included trials, 6 studies that reported the relationship between high PLR and OS were selected into this meta-analysis [24–26, 29, 31, 32]. Since the BCLC stages of HCC patients may influence OS significantly, we compared the relationship of PLR and OS among patients with different BCLC stages. The results indicated that in total BCLC stages, the heterogeneity of the high-PLR studies was 10%. Therefore, a random effects model was used for statistical analysis, and the result showed that patients with a high baseline PLR may have a lower OS rate (HR = 1.73; 95% CI: (1.46, 2.04); *P* < 0.00001) (Figure 2(a)). Sensitivity analyses suggested that the pooled effect of the PLR on OS was not affected by changing effect model. The funnel plot was symmetric (Figure 2(b)). For further analysis, we made a reasonable classification of the precise BCLC stage since several trials could not get specific count relationship between the PLR and corresponding BCLC stage in the OS. For example, the study of Ni et al. [32] was considered as BCLC-A subgroup with the larger proportion of BCLC-A patients. Finally, the included studies were divided into BCLC-A subgroup or BCLC-B/C subgroup. The prognostic role of high PLR for OS in different BCLC stages is shown in Figure 2(a). In the BCLC-A subgroup, the result indicated high statistical heterogeneity with an *I*^2^ value of 78% (HR = 1.54; 95% CI: (0.99, 2.39); *P* = 0.06). However, for the BCLC-B/C subgroup, 4 trials showed almost no heterogeneity in the consistency of the trial results (*I*^2^ = 2%). The prognostic role of high PLR for OS was favored with statistically significant (HR = 1.76; 95% CI: (1.47, 2.11); *P* < 0.00001).

### 3.5. Correlation between PLR and DFS

**Figure 3:**
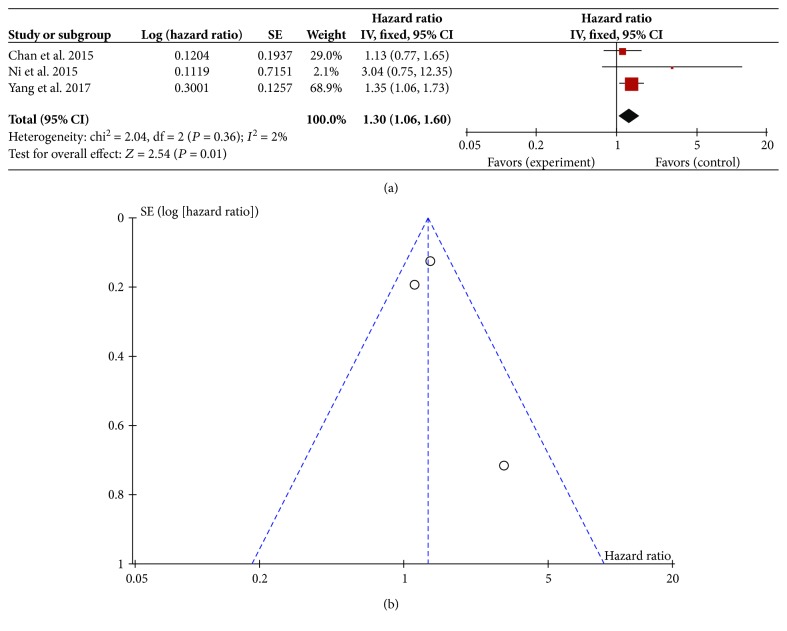
Correlation between platelet-to-lymphocyte ratio and disease-free survival. (a) Forest plot of comparison of the included trials; (b) funnel plot of comparison of the included trials.

As another index for the prognosis of HCC patients, DFS was reported in 3 studies [26, 31, 32]. Therefore, the relationship between PLR and DFS of these 3 included studies was analyzed. As shown in Figure 3(a), the result showed mild heterogeneity in the consistency of these trials (*I*^2^ = 2%). Therefore, a random effects model was used for meta-analysis, and the result indicated that patients with high pretreatment PLR had poor DFS (HR = 1.30; 95% CI: (1.06, 1.60); *P* = 0.01). Sensitivity analyses suggested that the pooled effect of PLR on DFS was not affected by changing effect model. The funnel plot was symmetric (Figure 3(b)).

### 3.6. Correlation between PLR and Event Occurrence

**Figure 4:**
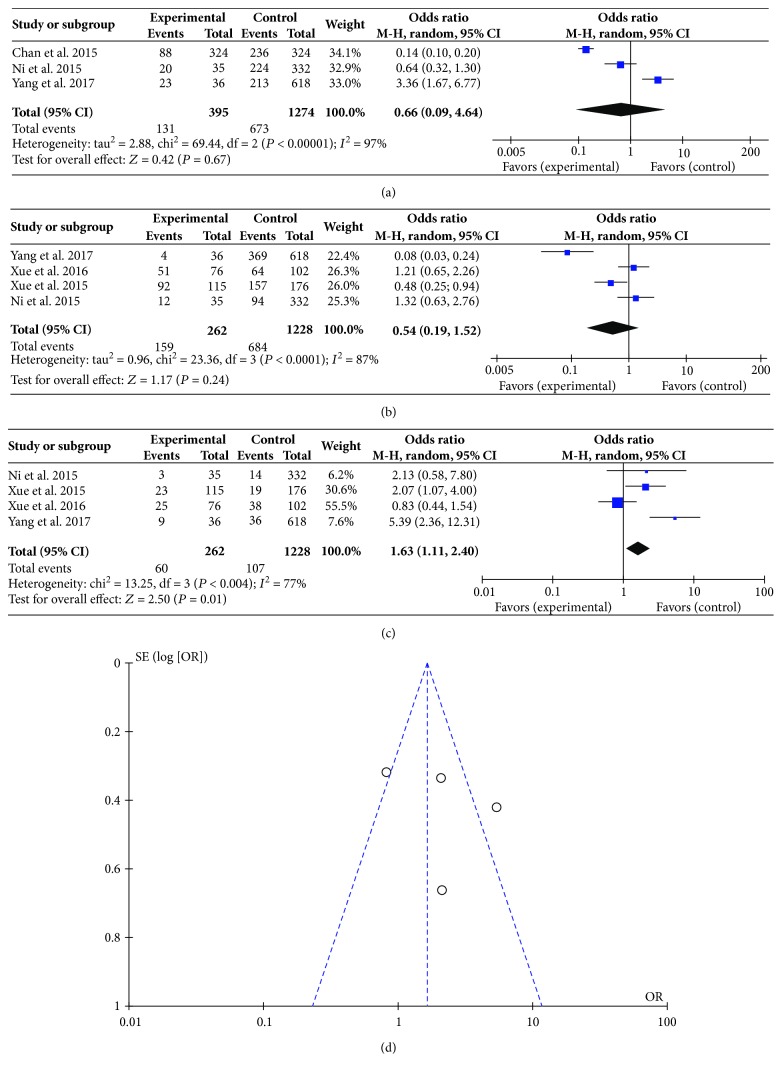
Correlation between platelet-to-lymphocyte ratio and event occurrence. (a) Forest plot of comparison of the included trials of BCLC-A; (b) forest plot of comparison of the included trials of BCLC-B; (c) forest plot of comparison of the included trials of BCLC-C; (d) funnel plot of comparison of the included trials of BCLC-C.

Apart from the main indexes such as OS and DFS observed in these trials, event occurrences such as total bilirubin and alanine transaminase were also indicated in 5 trials [24, 26, 29, 31, 32]. These studies investigated the correlation between PLR and event occurrence. Thus, we further analyze their relationship according to different BCLC stages. As shown in Figures 4(a) and 4(b), the heterogeneity in the results of BCLC-A and BCLC-B was high with an *I*^2^ value of 97% and 87%, respectively. And the test for overall effect indicated no statistical significance (*P* > 0.05). However, for the BCLC-C patients, although obvious heterogeneity was observed in the meta-analysis (*I*^2^ = 77%), the test for overall effect indicated statistical significance (*P* = 0.01), which meant that high PLR may be related to poor clinical event occurrence (Figure 4(c)). Sensitivity analyses suggested that the pooled effect of PLR on event occurrence was affected by changing random effects model. The funnel plot showed that 2 trials were out of the symmetric region (Figure 4(d)).

## 4. Discussion

As a major health problem, liver cancer is the sixth most common cancer worldwide with approximately 850,000 new cases diagnosed each year and the second leading cause of cancer-related deaths with approximately 800,000 death toll per year. Among all primary liver cancers, HCC constitutes 85–90% [33–35]. However, no standard quantitative biomarkers are perfect enough to assess the clinical outcomes in patients with HCC until now. Considering the reproducible and consistent features of biomarkers, blood parameters such as NLR and PLR may be potential since they are convenient and easy to be acquired during routine clinical practice.

The predicted role of PLR has been studied among various cancers. For example, Zhao et al. [36] evaluated the prognostic significance of PLR in esophageal cancer patients with a total of 6699 patients from 16 studies. The results demonstrated that higher PLR predicted poorer OS, DFS, and CFS. Elevated PLR is also negatively related to the OS of patients with urological cancers except bladder cancer [37].

In the present study, we utilized the existing evidence from 9 included studies to obtain the pooled results to evaluate the predicted role of PLR in HCC. The results showed that an elevated pretreatment PLR indicated unfavorable worse OS (HR = 1.73; 95% CI: (1.46, 2.04); *P* < 0.00001) and DFS (HR = 1.30; 95% CI: (1.06, 1.60); *P* = 0.01), although there are several studies that focused on the relationship between PLR and HCC prognosis. For example, Fu et al. retrospectively analyzed the data of 268 patients with operable solitary large HCC and found that PLR combined with microvascular invasion and tumor size could be considered as a score system to predict survival in solitary large HCC. Multivariate analysis showed that PLR is associated with DFS significantly (HR = 1.004, *P* = 0.003), indicating that PLR may be a potential prognostic index for patients with solitary large HCC [38]. The study of He and Lin [25] just focused on HCC patients treated with TACE and recombinant human type-5 adenovirus H101, while Lai et al. [39] focused on the role of PLR in HCC patients after liver transplantation. In spite that Fu et al. [40] investigated the prognostic value of PLR and BCLC stages among HCC patients who underwent hepatectomy, they just concluded that BCLC stage could be considered as an independent predictor of OS and RFS without the relationship of PLR and BCLC stages. In our current meta-analysis, inclusion criteria of every trial were HCC and BCLC staging criteria, not for TNM or other unclear staging criteria. We further analyzed the role of PLR in different BCLC stages. As for the correlation between PLR and OS, high PLR indicating poor OS in BCLC-B/C patients was statistically significant without heterogeneity in the consistency of the trial results, while the result of BCLC-A patients indicated high statistical heterogeneity with an *I*^2^ value of 78%. On the other hand, as for the correlation between PLR and event occurrence, high PLR was related to poor clinical event occurrence in BCLC-C patients only, while obvious heterogeneity was observed in all different BCLC stages. Therefore, more prospective cohort studies should be carried out to explore the prognostic role of PLR in different BCLC stages of HCC. Furthermore, we enrolled HCC patients with various treatments such as TACE, hepatectomy, and sorafenib, which may help to decrease the heterogeneity and extend the use of PLR in HCC patients.

It is also to be noted that several limitations of our study should be carefully considered. Firstly, all the enrolled studies were retrospective, thus some biases, such as information bias, misclassification bias, and selection bias, may have existed in the meta-analysis. Secondly, the sample size in the present study is so small that only 3 trials were enrolled in the analysis of the correlation between PLR and DFS. Thirdly, to unify the statistical method, we adopt univariable HR that might also increase bias into our study. Fourthly, no study mentioned information regarding dropouts, which might have exaggerated the prognostic effects. Finally, platelet and lymphocyte levels are easily influenced by other factors, such as infection, inflammation in other tissues, and medications taken before HCC treatment, and thereby the PLR measurement may be affected.

Taking all of these into consideration, PLR may be considered as a significant biomarker in the prognosis of HCC in different BCLC stages. Compared to other prognostic markers, PLR seems to be an inexpensive, widely-obtained, repeatable, and reliable predictor for HCC patients. HCC patients with high PLR may benefit from modifying inflammatory responses and modulating the immune system. More studies are warranted to draw a more powerful conclusion that may be significant for clinical practice.

## Figures and Tables

**Table 1:** Characteristics of the included studies.

Study	Year	Country	Treatment	Study time	Sample size	Mean age (y)	Male	PLR cutoff value	Measurement index	Follow-up (median; months)	BCLC stage of patients
He and Lin et al.	2017	Chinese	TACE	2007.1–2015.7	216	53.05	200	94.62	NLR, PLR, PNI, PI, mGPS, NLR-PLR	14.4	A1 (23); A2 (4); A4 (9); B (98); C (82)
Yang et al.	2017	Chinese	Hepatectomy	2010.4–2013.10	778	51.98	671	150	PLR	NR	0/A (236); B/C (537)
Liu et al.	2016	Chinese	Hepatectomy	2004.7–2011.4	223	54	189	NR	PLR, NLR, APRI	NR	0/A (126); B/C (97)
Casadei et al.	2016	Italy	Sorafenib	2012–2015	56	NR	47	15.0	PLR, NLR, SII	NR	B (13); C (43)
Xue et al.	2016	Chinese	TACE	2007.1–2011.4	178	52.57	154	150	PLR	11.4	B (115); C (63)
Shiozawa et al.	2016	Japan	Sorafenib	2009.6–2015.1	16	67.5	12	ΔPLR% > 20%	NLR, PLR	NR	B (12); C (4)
Chan et al.	2015	Chinese	Hepatectomy	2001.1–2011.11	324	56.8	283	150	PLR, NLR, PNI	NR	A (324)
Xue et al.	2015	Chinese	TACE	2007.1–2011.3	291	53.05	258	150	PLR, NLR, PNI	NR	B (182); C (109)
Ni et al.	2015	Chinese	Hepatectomy	2010.12–2012.1	367	55	308	150 and 300	GPS, mGPS, NLR, PLR, PI, PNI	24	A (244); B (106); C (17)

TACE: transarterial chemoembolization; NR: not reported; NLR: neutrophil-to-lymphocyte ratio; PLR: platelet-to-lymphocyte ratio; PNI: prognostic nutritional index; PI: prognostic index; mGPS: modified Glasgow Prognostic Score; NLR-PLR: neutrophil/platelet-to-lymphocyte ratio; APRI: aspartate aminotransferase/platelet ratio index; SII: systemic immune-inflammation index; GPS: Glasgow Prognostic Score; PI: prognostic index.

**Table 2:** Quality assessment and risk of bias of the included trials.

Study	Selection				Comparability	Exposure			Score
	1	2	3	4	5	6	7	8	
He and Lin	^∗^	^∗^			^∗^	^∗^	^∗^	^∗^	6
Yang et al.	^∗^	^∗^			^∗^	^∗^	^∗^	^∗^	6
Liu et al.	^∗^	^∗^				^∗^	^∗^	^∗^	5
Casadei et al.	^∗^	^∗^			^∗^	^∗^	^∗^	^∗^	6
Xue et al.	^∗^	^∗^			^∗^	^∗^	^∗^	^∗^	6
Shiozawa et al.	^∗^	^∗^			^∗^	^∗^	^∗^	^∗^	6
Chan et al.	^∗^	^∗^			^∗^	^∗^	^∗^	^∗^	6
Xue et al.	^∗^	^∗^			^∗^	^∗^	^∗^	^∗^	6
Ni et al.	^∗^	^∗^			^∗^	^∗^	^∗^	^∗^	6

^∗^The score of each item.
